# The anti‐cancer effect of epigallocatechin‐3‐ *O*‐gallate against multiple myeloma cells is potentiated by 5,7‐dimethoxyflavone

**DOI:** 10.1002/2211-5463.13708

**Published:** 2023-09-25

**Authors:** Jaehoon Bae, Motofumi Kumazoe, Su‐Jin Park, Yoshinori Fujimura, Hirofumi Tachibana

**Affiliations:** ^1^ Division of Applied Biological Chemistry, Department of Bioscience and Biotechnology, Faculty of Agriculture Kyushu University Fukuoka Japan; ^2^ Functional Biomaterial Research Center Korea Research Institute of Bioscience and Biotechnology Jeongeup‐si Korea

**Keywords:** (−)‐epigallocatechin‐3‐*O*‐gallate, 5,7‐dimethoxyflavone, acid sphingomyelinase, cyclic guanosine monophosphate, multiple myeloma

## Abstract

(−)‐Epigallocatechin‐3‐*O*‐gallate (EGCG) is one of the major components of green tea polyphenol. Previous studies have shown that EGCG induces cancer‐specific cell death *in vitro* and *in vivo* without causing severe side effects. However, the anti‐cancer effect of EGCG alone is limited. 5,7‐dimethoxyflavone (5,7‐DMF), one of the principal functional components of black ginger (*Kaempferia parviflora*), also exerts anti‐cancer effects. Here, we show that 5,7‐DMF synergistically enhances the anti‐cancer effect of EGCG in multiple myeloma cells by potentiating EGCG‐induced intracellular cyclic guanosine monophosphate (cGMP) production. Moreover, the combination of EGCG and 5,7‐DMF induces apoptotic cell death in multiple myeloma cells, and this is accompanied by activation of the cGMP/acid sphingomyelinase (ASM)/cleaved caspase‐3 pathway. In conclusion, we have shown that 5,7‐DMF enhances the anti‐cancer effect of EGCG by upregulating cGMP in multiple myeloma cells.

## Graphical abstract

Black ginger (*Kaempferia parviflora*)‐derived 5,7‐dimethoxyflavone (5,7‐DMF) synergistically enhances the anti‐cancer effect of green tea polyphenol (−)‐Epigallocatechin‐3‐*O*‐gallate (EGCG) in multiple myeloma cells. This enhancement is accompanied by activation of the cyclic guanosine monophosphate (cGMP)/acid sphingomyelinase/cleaved caspase‐3 pathway. In conclusion, we have shown that 5,7‐DMF enhances the anti‐cancer effect of EGCG by upregulating cGMP in multiple myeloma cells.

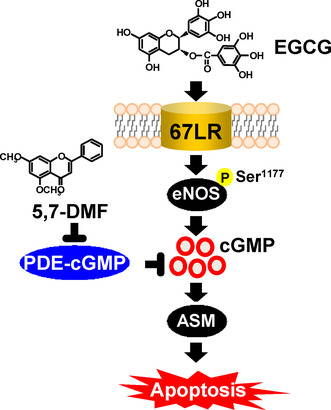

**Abbreviations**
ASM: acid sphingomyelinase
cGMP: cyclic guanosine monophosphate
EGCG: (−)‐Epigallocatechin‐3‐*O*‐gallate
IC_50_
: 50% inhibitory concentrations
NO: nitric oxide
PDEs: phosphodiesterases
sGC: soluble guanylate cyclase
5,7‐DMF: 5,7‐dimethoxyflavone
67LR: 67‐kDa laminin receptor

Green tea, and one of its major components polyphenol, (−)‐epigallocatechin‐3‐*O*‐gallate (EGCG), have been shown to have cancer‐therapeutic effects [1, 2, 3]. Previously, we identified 67‐kDa laminin receptor (67LR) as the sensing molecule for EGCG [4, 5, 6]. 67LR is overexpressed in numerous types of cancer cells, including multiple myeloma [6, 7, 8, 9, 10]. Indeed, EGCG selectively induced apoptosis in cancer cells without affecting normal cells by targeting 67LR [6, 7, 8, 9]. EGCG induces apoptosis in several cancer cells, including multiple myeloma, pancreatic cancer, and prostate cancer cells via upregulating cyclic guanosine monophosphate (cGMP) dependent mechanisms [6]. These results suggested that EGCG induces anti‐cancer activity and provide a reasonable basis for its evaluation in clinical trials [9, 10, 11]. However, the effect of EGCG at physiological concentration is not enough to induce a sufficient anti‐cancer effect.

cGMP is a second messenger that processes several physiological effects, including anti‐cancer effects [6]. Intracellular cGMP production is increased by soluble guanylate cyclase (sGC), the receptor for nitric oxide (NO) [6, 12, 13, 14, 15, 16, 17].

Cyclic nucleotide phosphodiesterases (PDEs) play a pivotal role in cGMP‐ or/and cAMP‐mediated cell signaling and are well known as inactivators of cGMP or/and cAMP in mammalian tissues. PDE5 is one of the cGMP‐specific enzymes that degrade intracellular messenger cGMP production [18, 19, 20, 21]. Recently, it has been shown that inhibition of PDE enzyme activity might be a new therapeutic approach for various pathologies [18, 19, 20]. PDE5 inhibitors have shown positive effects for numerous pathologies, including erectile dysfunction [22]. Moreover, inhibition of PDE5 has shown to enhance cGMP‐dependent cancer apoptosis pathway in several cancer cells [6, 7].

Black Ginger (*Kaempferia parviflora*), also known as black turmeric, contains various types of flavonoids. Moreover, it contains a high amount of polymethoxtflavone among the flavonoids. 5,7‐dimethoxyflavone (5,7‐DMF) present is one of the major polymethoxyflavones in black ginger and is the potent PDE5 inhibitor [23]. Also, several studies have demonstrated that 5,7‐DMF induces various health‐promoting effect, including anti‐cancer effects [24, 25, 26].

In this study, we showed that 5,7‐dimethoxyflavone synergistically potentiated the anti‐cancer effect of EGCG, accompanied by the enhancement of EGCG‐induced cGMP upregulation in multiple myeloma cells.

## Materials and methods

### Cell culture

5,7‐DMF was purchased from Wako (Tokyo, Japan). Bay 41‐2272 was obtained from Enzo Life Sciences (Exeter, Devon, UK). EGCG, catalase, and superoxide dismutase (SOD) were obtained from Sigma‐Aldrich (St Louis, MO, USA). The human multiple myeloma cell line U266, at a density of 5 × 10^4^ cells·mL^−1^, was cultured in RPMI 1640 supplemented with 1% (v/v) fetal bovine serum, 200 U·mL^−1^ catalase, and 5 U·mL^−1^ superoxide dismutase (SOD) from Sigma‐Aldrich at 37 °C, 100% humidity, and 5% CO_2_. After 96 h, cell viability was determined using trypan blue staining assay.

### Analyzed apoptotic cell death

2 × 10^4^ cells·mL^−1^ of U266 cells were cultured in 1% FBS‐RPMI 1640 at 100% humidity and 5% CO_2_, at 37 °C. Apoptotic cells were determined using a flow cytometric test. Annexin‐V^+^ cells were evaluated by combining early Annexin‐V^+^ propidium iodide‐ and late Annexin‐V^+^ propidium iodide_+_ cells after treatment with EGCG or/and 5,7‐DMF. The analysis was performed after 96 h using a Verse™ system from BD (Bergen county, NJ, USA).

### Western blot analysis

U266 cells were seeded onto a 12‐well plate at a density of 1 × 10^6^ or 5 × 10^4^ cells per well and treated with 5 μm EGCG or/and 10 μm 5,7‐DMF for 3 or 96 h. After treatment, cells were lysed in a solution containing 50 mm Tris–HCl (pH 7.5), 150 mm NaCl, 1% Triton X‐100, 2 mg·mL^−1^ aprotinin, 50 mm NaF, 1 mm phenylmethanesulfonyl fluoride, 30 mm Na_4_P_2_O_7_, 1 mm pervanadate, and 1 mm ethylenediaminetetraacetic acid. SDS/PAGE (sodium dodecyl sulphate‐polyacrylamide gel electrophoresis) was performed as previously described [9]. Approximately 50 μg of protein was suspended in Laemmli sample buffer (0.1 m Tris–HCl buffer, pH 6.8; 0.05% mercaptoethanol; 1% SDS; 0.001% bromophenol blue; and 10% glycerol) and boiled before electrophoresis on SDS‐polyacrylamide gels. The gels were then transferred to Trans‐Blot nitrocellulose membranes from Bio‐Rad (Berkeley, CA, USA) using electroblotting. Primary antibodies were incubated in Tween 20‐TBS containing 1% BSA. The blots were washed with Tween 20‐TBS and then incubated in anti‐rabbit HRP conjugates (secondary antibody). The primary antibody used at a 1 : 3000 dilution was incubated overnight at 4 °C, followed by the secondary antibody at a 1 : 10 000 dilution for 1 h. The anti‐eNOS (H‐159) antibody was purchased from Santa Cruz Biotechnology (Dallas, TX, USA), the anti‐P‐eNOS (Ser1177) antibody was obtained from BD Biosciences (San Diego, CA, USA), the anti‐Cleaved caspase‐3 antibody was obtained from Cell Signaling Technology (Danvers, MA, USA), the anti‐Laminin‐R (MLuC5) antibody was purchased from Santa Cruz Biotechnology, the anti‐IgM (A‐7) antibody was purchased from Santa Cruz Biotechnology, the anti‐67 kDa Laminin Receptor (RPSA/2699) antibody was purchased from Abcam (Cambridge, UK), and the anti‐β actin antibody was purchased from Sigma‐Aldrich.

### Quantitative reverse transcription PCR (qRT‐PCR)

U266 cells were cultured with the presence of 5 μm EGCG or/and 10 μm 5,7‐DMF. After 3 h, the culture medium was removed, and the cells were washed two times with PBS. After then, cells were collected by centrifugation at 500 ***g*** for 5 min. Total RNA was isolated using the Qiagen RNeasy Mini Kit from QIAGEN (Hilden, North rhin‐westphalia, Germany) according to the manufacturer's instructions. Primers were used for homo sapiens ribosomal protein SA (RPSA, 67LR), Forward: 5′‐GCAGCAGGAACCCACTTAGG‐3′, Reverse: 5′‐GCAGCAGCAAACTTCAGCAC‐3′; Human GAPDH primer, Forward: 5′‐CCACTCCTCCACCTTTGACG‐3′ (upstream), Reverse: 5′‐CCACCACCCTGTTGCTGTAG‐3′. cDNA was synthesized from total RNA using the cDNA Master Mix from Applied Biosystems (Los Angeles, CA, USA). qRT‐PCR was conducted using 2 μL cDNA and SYBR Green PCR 2 × Master Mix from Applied Biosystems with 40 cycles of 15 s at 95 °C, 60 s at 60 °C according to the manufacturer's instructions. The data were analyzed using v2.1 stepone software from Applied Biosystems.

### cGMP assays

Measurements of intracellular cGMP production were carried out using the TRFRET cGMP assay kit (Perkin‐Elmer) following the manufacturer's protocol. The cells were treated with EGCG and/or 5,7‐DMF for 3 h in 96‐well plates, and the plate assessment was performed using the EnVision™ Plate Reader from Perkin‐Elmer (Waltham, MA, USA).

### ASM activity measurement

U266 cells were lysed with lysis buffer and incubated for 1 h at 4 °C, followed by centrifugation for 20 min at 15 000 ***g***. The resulting supernatant was incubated with substrate buffer, including 200 mm sodium acetate, 1% Triton X‐100, and 400 pmol BODIPY‐C12 sphingomyelin in dH_2_O, for 18 h at 37 °C. BODIPY‐C12‐sphingomyelin was obtained from Sigma‐Aldrich.

### Statistical analysis

Our data are indicated as mean ± SEM. Calculation the IC_50_ values and isobologram methods were determined by using the graphpad software (San Diego, CA, USA). Tukey's test was used to assess the significance of differences between experimental variables. Statistical analyses were performed using kyplot software (Kyens Lab, Tokyo, Japan).

## Results

### 5,7‐DMF synergistically potentiates cell death effect of EGCG in U266 cells

**Fig. 1:**
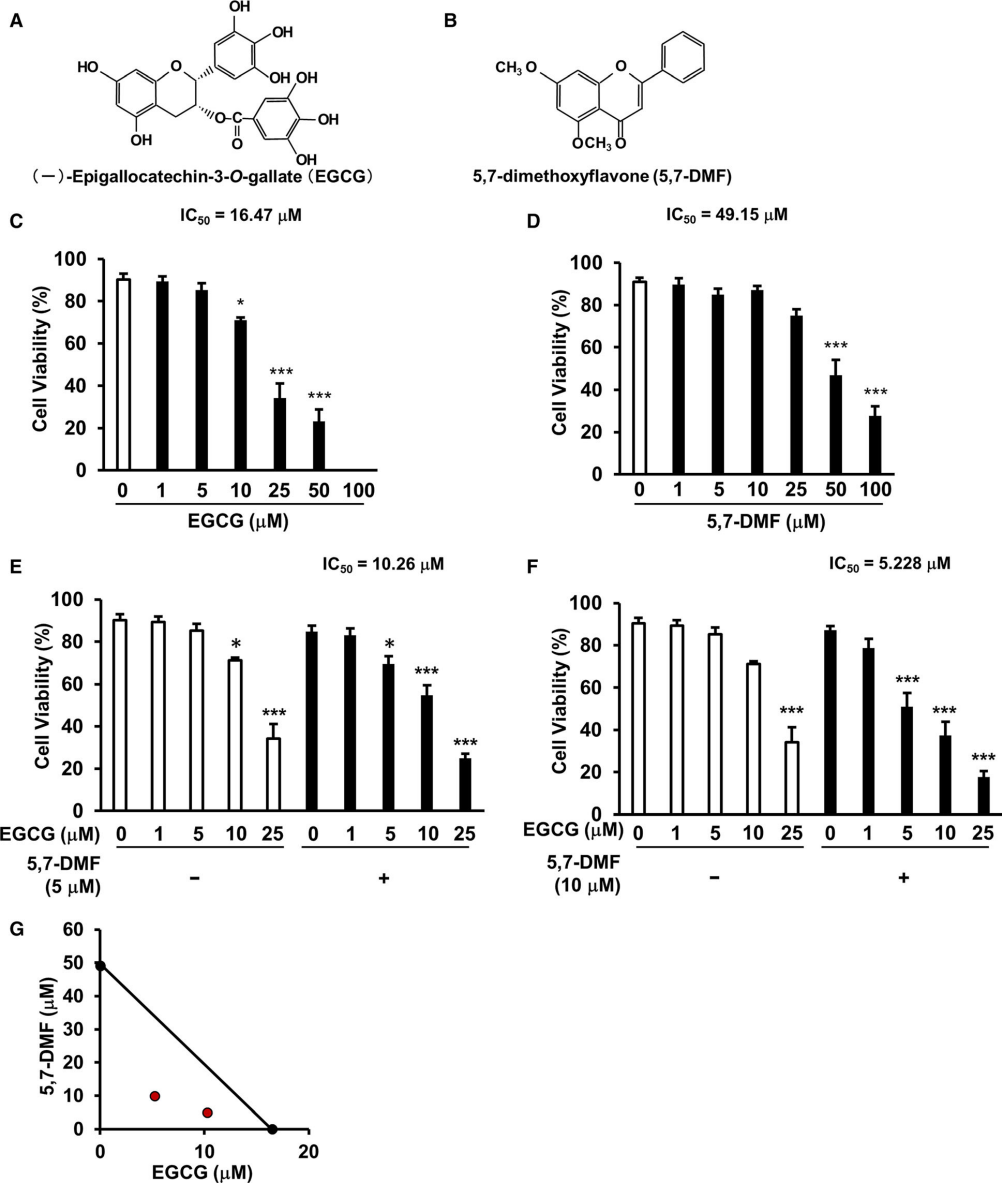
5,7‐DMF synergistically potentiates cell death effect of EGCG in U266 cells. (A, B) Based on chemical structure of EGCG and 5,7‐DMF. (C–F) The human multiple myeloma cell line U266 was cultured with or without 5,7‐DMF and/or EGCG at the indicated concentration for 96 h, and the viability of U266 was measured by trypan blue staining assay. (G) The combination effect of EGCG and 5,7‐DMF was measured by isobologram analysis in U266 cells. Data are presented as mean ± SEM (*n* = 3), Tukey's test, *P < 0.05, ***P < 0.001.

We showed based on the chemical structure of EGCG and 5,7‐DMF (Fig. 1A,B). EGCG selectively induced apoptosis in cancer cells by targeting the 67LR [8, 9, 27]. However, the physiological concentration of EGCG is limited in this effect [28]. We assessed the anti‐multiple myeloma effect of the combination of 5,7‐DMF and EGCG in U266 cells. We showed 5 or 10 μm 5,7‐DMF significantly potentiated the multiple myeloma killing activity of EGCG, with 50% inhibitory concentrations (IC_50_) values of 10.26 or 5.228 μm, while the IC_50_ values for EGCG were 16.47 μm, and for 5,7‐DMF were 49.15 μm in U266 cells (Fig. 1C−F). Isobologram analysis is a well‐known analysis for evaluating synergy effects based on the dose–response manner of each drug. A straight line was observed between the *y*‐axis (representing 5,7‐DMF at 49.15 μm) and *x*‐axis (representing EGCG at 16.47 μm), respectively, on plotting the IC50 values on each axis. The isobologram plot of cell viability curves showed that the combination of EGCG and 5,7‐DMF was not just additive but had a synergistic effect in U266 cells (Fig. 1G).

### Combination of EGCG and 5,7‐DMF induces apoptotic cell death

**Fig. 2:**
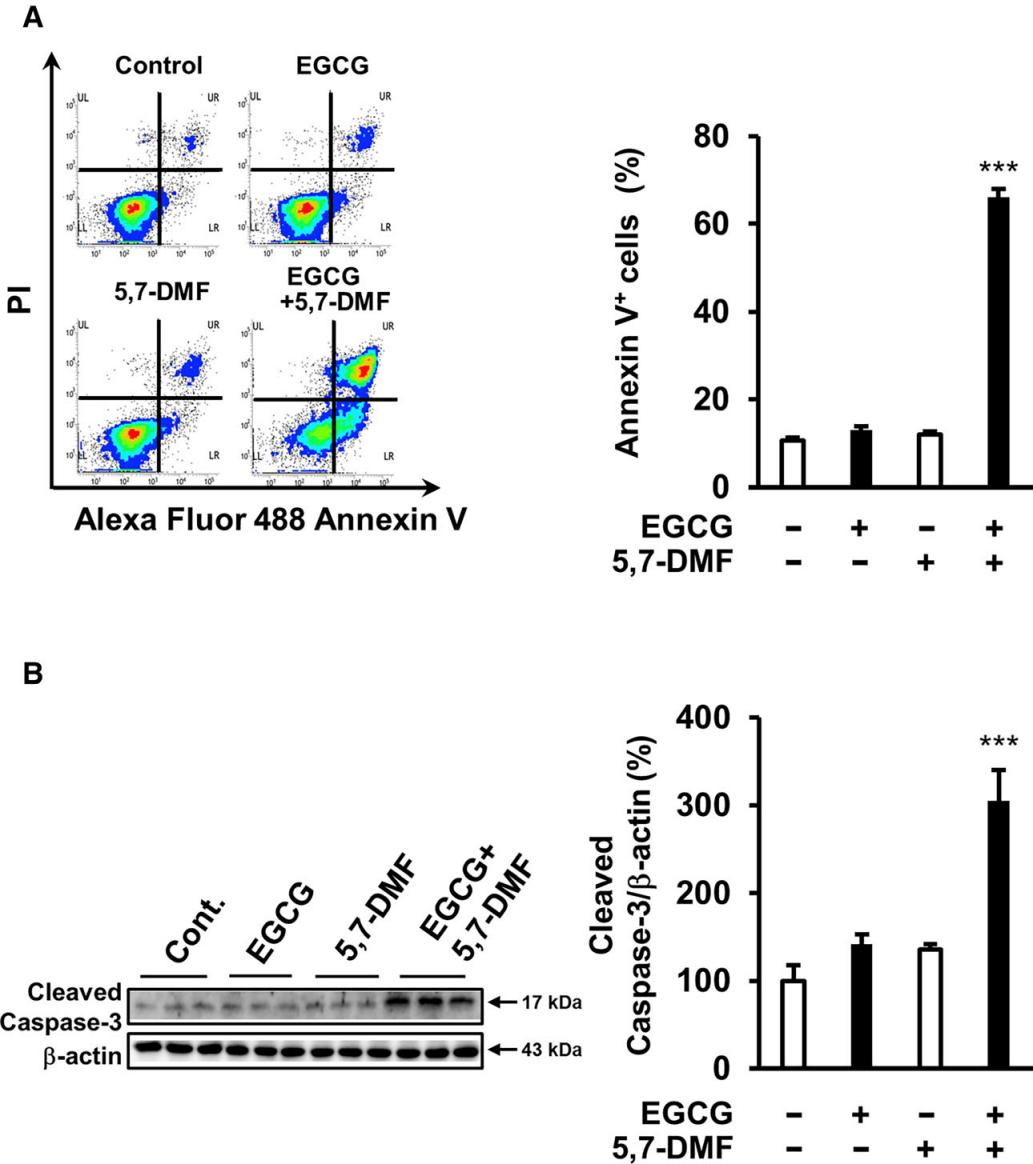
Combination of EGCG and 5,7‐DMF induces apoptotic cell death. (A) Apoptotic cells were stained with Annexin V–Alexa Fluor 488 and PI in U266 cells. (B) Cells were treated with or without EGCG (5 μm) in the presence or absence of 5,7‐DMF (10 μm) for 96 h, and cleaved caspase‐3 levels were assessed using western blot analysis. Data are presented as mean ± SEM (*n* = 3), Tukey's test, ***P < 0.001.

EGCG has been shown to induce apoptosis in multiple myeloma cells [6, 9]. To investigate whether the combination of EGCG and 5,7‐DMF induces apoptosis, we treated U266 cells with EGCG and/or 5,7‐DMF. Our results demonstrate that 10 μm 5,7‐DMF significantly potentiated the apoptosis‐inducing effect of EGCG, whereas treatment with 5 μm EGCG or 10 μm 5,7‐DMF alone did not induce apoptosis in U266 cells (Fig. 2A). Additionally, we found that the level of cleaved caspase‐3, a key mediator of apoptosis, increased significantly in U266 cells treated with the combination of EGCG and 5,7‐DMF (Fig. 2B). Taken together, these results suggested that the 5,7‐DMF potentiates apoptosis inducing effect of EGCG in U266 cells.

### 5,7‐DMF potentiates anti‐cancer effect of cGMP inducer bay 41‐2272

**Fig. 3:**
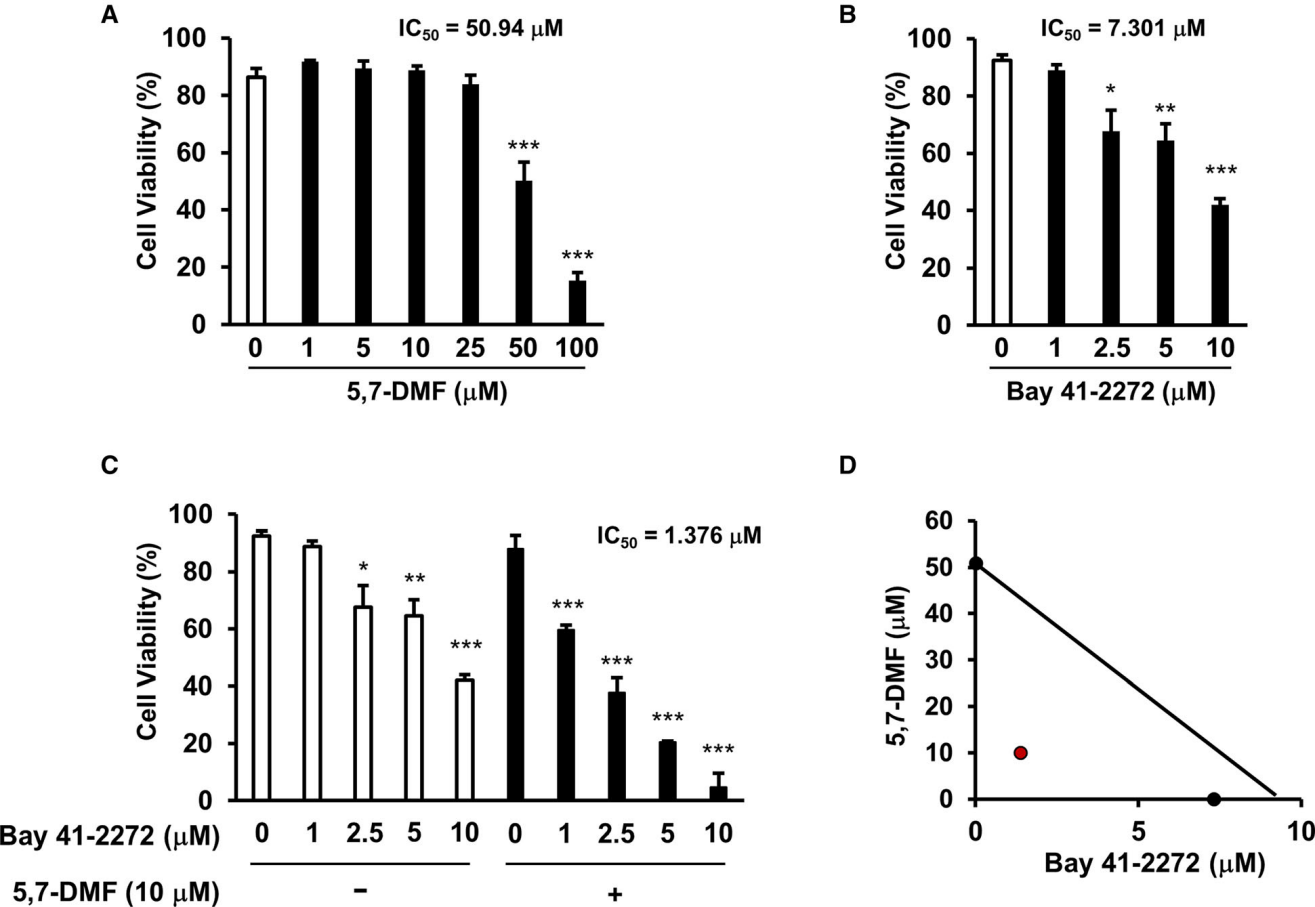
5,7‐DMF potentiates intracellular cGMP activator Bay 41‐2272‐induced apoptotic cell death. (A–C) U266 cells were cultured with/without 5,7‐DMF and/or Bay 41‐2272 at the indicated concentration for 96 h, and viability was measured by trypan blue staining assay. (D) The synergy effect of Bay 41‐2272 and 5,7‐DMF is measured by isobologram analysis. Data are presented as mean ± SEM (*n* = 3), Tukey's test, *P < 0.05, **P < 0.01, ***P < 0.001.

To estimate whether intracellular cGMP production is sufficient to induce cell death in multiple myeloma, we assessed the effect of 5,7‐DMF and/or Bay 41‐2272 in U266 cells (Fig. 3). We found that 5,7‐DMF did not induce cell death at concentrations ranging from 0 to 25 μm (Fig. 3A). Bay 41‐2272 induced cell death in a dose‐dependent manner in U266 cells, with IC50 values for Bay 41‐2272 of 7.301 μm (Fig. 3B). Moreover, we demonstrated that 5,7‐DMF potentiated Bay 41‐2272‐induced cell death, with IC50 values of 1.376 μm (Fig. 3C). Isobologram analysis of cell viability curves showed that the combination effect of EGCG and Bay 41‐2272 was not just additive but was synergistic (Fig. 3D). These results suggested that 5,7‐DMF enhanced cGMP‐inducer induced cell death in U266 cells.

### 5,7‐DMF potentiates EGCG‐induced apoptotic cell death through activation of cGMP pathway

**Fig. 4:**
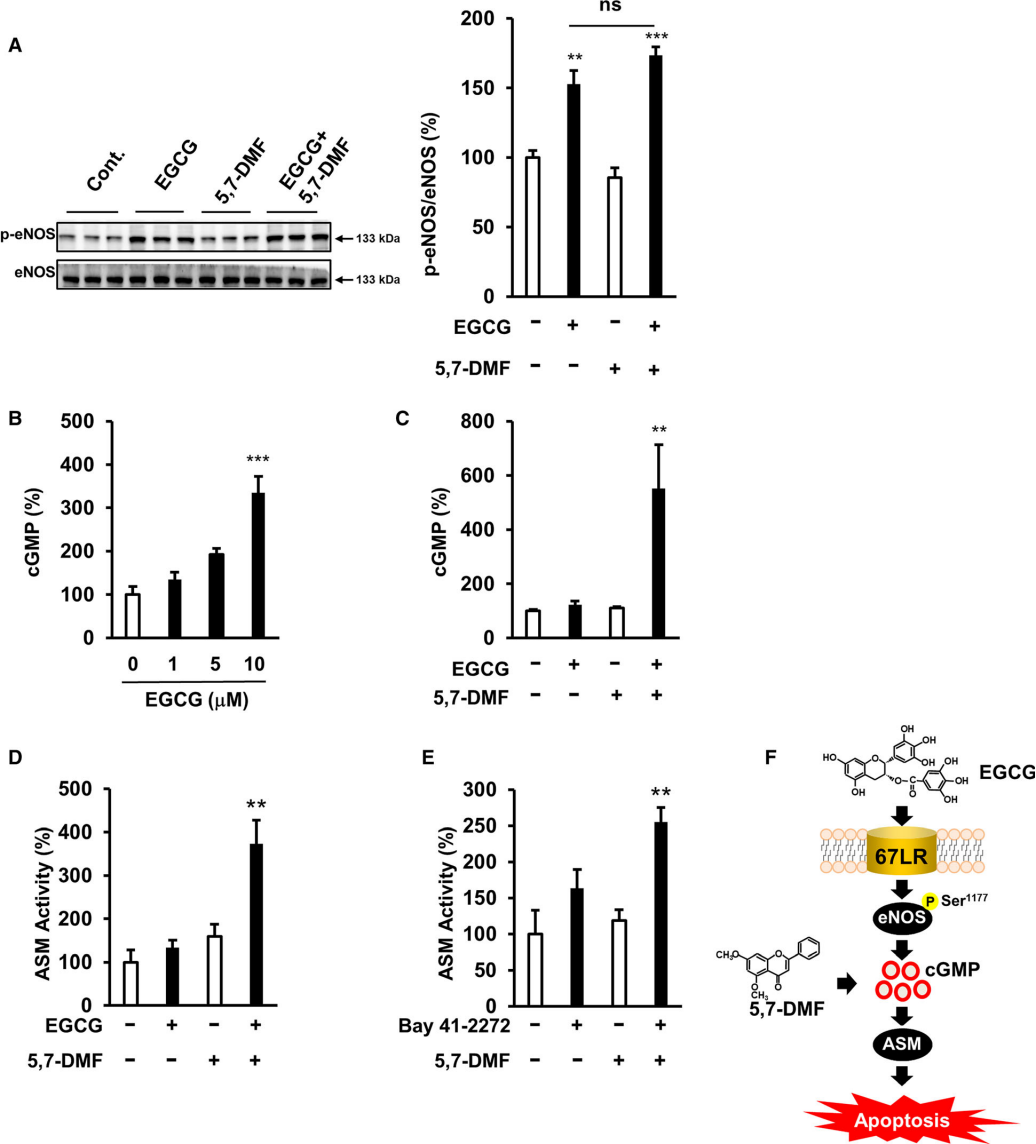
5,7‐DMF potentiates EGCG‐induced apoptotic cell death through activation of cGMP pathway. (A) U266 cells were treated with 5 μm EGCG and/or 10 μm 5,7‐DMF for 3 h, and eNOS phosphorylation at Ser1177 was assessed using western blot analysis (*n* = 3). (B) Intracellular cGMP production induced by EGCG was evaluated in U266 cells (*n* = 4). Cells were treated with EGCG for 3 h, and intracellular cGMP production was determined. (C) Intracellular cGMP production was determined in cells treated with 5 μm EGCG or/and 10 μm 5,7‐DMF for 3 h (*n* = 4). (D) ASM activity was determined by TLC analysis in cells treated with 5 μm EGCG or/and 10 μm 5,7‐DMF (*n* = 3). (E) ASM activity was assessed using TLC analysis in cells treated with 1 μm Bay 41‐2272 or/and 10 μm 5,7‐DMF (*n* = 3). (F) The schematic diagram shows the signaling pathways of EGCG and 5,7‐DMF in multiple myeloma cells. Data are presented as mean ± SEM (*n* = 3). Tukey's test, **P < 0.01, ***P < 0.001.

To evaluate the effect of 5,7‐DMF on the upstream signal of cGMP, we determined its effect on 5,7‐DMF on EGCG‐derived eNOS phosphorylation. We observed that EGCG induced eNOS phosphorylation at Ser1177, but 5,7‐DMF did not affect it (Fig. 4A). We showed that 10 μm EGCG induced cGMP production in U266 cells, while 0–5 μm EGCG did not have a significant effect (Fig. 4B). Moreover, our data showed that 5,7‐DMF potentiated the EGCG‐induced cGMP production in U266 cells (Fig. 4C). These finding suggested that 5,7‐DMF may reduce the cGMP‐PDE enzyme activity in U266 cells. We showed 5,7‐DMF synergically potentiated the ASM activity of EGCG (Fig. 4D). To determine the effect of 5,7‐DMF on cGMP‐dependent signaling in multiple myeloma cell death, we assessed its effect on the BAY 41‐2272‐induced activation of ASM, which is known to be downstream of cGMP. 5,7‐DMF potentiated BAY 41‐2272‐elicited activation of ASM in U266 cells (Fig. 4E). We showed a schematic representation indicating that 5,7‐DMF potentiates the EGCG‐induced apoptotic cell death in human multiple myeloma U266 cells (Fig. 4F).

Taken together, these results suggested that 5,7‐DMF potentiates EGCG‐induced cell death in multiple myeloma cells, accompanied by an enhancement of cGMP‐dependent multiple myeloma cell death signaling in U266 cells.

### 5,7‐DMF enhanced anti‐cancer effect of EGCG through 67LR without affecting expression levels of 67LR

**Fig. 5:**
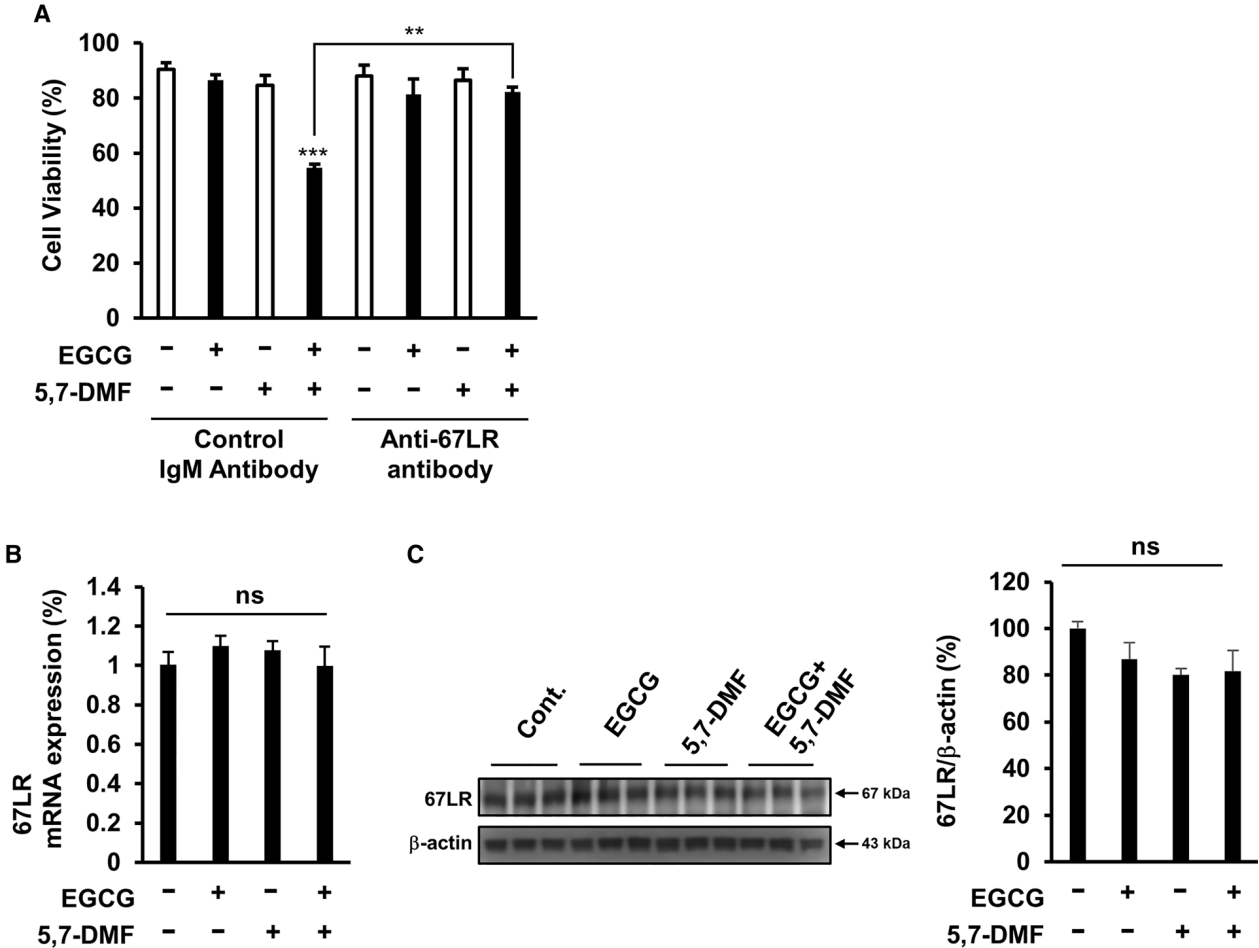
5,7‐DMF induced a cell death effect of EGCG through 67LR without affecting expression levels of 67LR. (A) U266 cells were treated with control IgM antibody or anti‐67LR antibody (MLuC5) for 2 h and then the cells were added to 5 μm EGCG and/or 10 μm 5,7‐DMF for 96 h. Cell viability was assessed by trypan blue staining assay (*n* = 3). (B) U266 cells were treated with 5 μm EGCG and/or 10 μm 5,7‐DMF for 3 h, and 67LR mRNA expression level was assessed using real‐time PCR (*n* = 3). (C) U266 cells were treated with 5 μm EGCG and/or 10 μm 5,7‐DMF for 3 h, and 67LR protein level was measured using western blot analysis (*n* = 3). Data are presented as mean ± SEM (*n* = 3), Tukey's test, **P < 0.01, ***P < 0.001.

Because anti‐67LR antibody treatment is widely used to assess the role of 67LR in EGCG‐induced cell death [6], we assess the role of 67LR in the anti‐cancer effect of the combination by using anti‐67LR antibody. Our result showed that anti‐67LR antibody pretreatment significantly attenuated the effect of the combination (Fig. 5A). Those results showed that 67LR is involved in the anti‐cancer effect of the combination. Moreover, we assessed the effect of EGCG/5,7‐DMF on the expression level of 67LR. Our result showed that the EGCG and/or 5,7‐DMF did not affect the 67LR expression levels (mRNA/protein; Fig. 5B,C). Taken together, these results suggested that the combination of EGCG and 5,7‐DMF induced cell death through 67LR without affecting expression levels of 67LR.

## Discussion

cGMP is a second messenger that involved in penile erection and vascular homeostasis. Clinical studies have shown that several direct or indirect intracellular cGMP producers are effective in treating many types of diseases, such as pulmonary hypertension, erectile dysfunction, and heart failure [19, 20, 21, 22]. Interestingly, EGCG‐induced cGMP plays a key role in inducing cell death in several types of cancer, including multiple myeloma cells [6]. However, the effect of EGCG at physiological concentrations is limited [28]. Here we demonstrated that 5,7‐DMF synergistically potentiates EGCG‐induced apoptotic cell death accompanied with intracellular cGMP production at physiological concentrations of EGCG in U266 cells.

Previous study has reported the anti‐cancer effects of 5,7‐DMF, a component derived from black ginger (*K. parviflora*) [26]. 5,7‐DMF from black ginger acts as an inhibitor of PDE5 activity, a specific negative regulator of cGMP [23, 29]. However, the concentrations of 5,7‐DMF used in that study were relatively high, with an IC50 value of 25 μm.

We showed that a single treatment of 5 μm EGCG or 10 μm 5,7‐DMF did not affect the cGMP level, while the combination of 5 μm EGCG and 10 μm 5,7‐DMF upregulated intracellular cGMP production in multiple myeloma cells. These results suggest that 5,7‐DMF potentiates EGCG‐induced multiple myeloma cell death accompanied with enhancing the cGMP induction activity of EGCG.

Nitric oxide (NO) is produced by NOS, followed by NO regulates sGC to induce intracellular cGMP levels [12, 13, 14, 15]. Previously, we assessed the role of NO/cGMP signaling activation in EGCG‐induced apoptosis mechanisms in cancer cells [6, 30]. We also showed that EGCG increased NO production in cancer cells including multiple myeloma cells [6]. EGCG also increased phosphorylation levels of eNOS at Ser1177, which upregulates its enzyme activity [6, 30]. We confirmed that EGCG induced eNOS phosphorylation at Ser1177, while a single treatment of 5,7‐DMF did not appear to induce eNOS phosphorylation at Ser1177. These results suggest that 5,7‐DMF potentiates EGCG‐induced multiple myeloma cell death by enhancing the downstream effects of NO, but it does not affect the upstream effects of NO, such as phosphorylation of eNOS at Ser1177 in U266 cells.

Previously, we reported that activation of ASM is a major downstream mechanism of EGCG‐induced cell death, including multiple myeloma cells [9]. This ASM activation is involved in several mechanisms of cancer cell apoptosis induced by cisplatin, oxidative stress, and ultraviolet light [31, 32]. ASM activation is an indispensable mediator in cGMP‐initiated apoptosis [6, 9]. Here, we showed that 5,7‐DMF potentiated the multiple myeloma‐killing activity of EGCG at physiological concentrations, accompanied with the activation of the cGMP/ASM/cleaved caspase‐3 pathway. We suggested that the cGMP/ASM mechanisms may contribute to these effects.

Taken together, we suggested that this mechanism and our using concentrations are worthy of particular consideration. Moreover, in combination of EGCG and 5,7‐DMF have high potential and could be effective for chemotherapy for treating multiple myeloma.

## Conflict of interest

The authors declare no conflict of interest.

### Peer review

The peer review history for this article is available at https://www.webofscience.com/api/gateway/wos/peer‐review/10.1002/2211‐5463.13708.

## Author contributions

JB, MK, YF, and HT designed the experiments; JB performed the experiments; JB and MK analyzed the data, wrote and revised the manuscript; YF and MK helped to organize figures, MK, YF, S‐JP and HT reviewed the manuscript. All authors contributed to the article and approved the final version submitted for publication.

## Data Availability

The original contributions presented in this study are included in the article material, and further inquiries can be directed to the corresponding author.
